# Cysteine Cathepsins and Their Extracellular Roles: Shaping the Microenvironment

**DOI:** 10.3390/cells8030264

**Published:** 2019-03-20

**Authors:** Eva Vidak, Urban Javoršek, Matej Vizovišek, Boris Turk

**Affiliations:** 1 Jozef Stefan Institute, Department of Biochemistry and Molecular and Structural Biology, Jamova 39, SI-1000 Ljubljana, Slovenia; eva.vidak@ijs.si (E.V.); urban.javorsek@ijs.si (U.J.); 2 International Postgraduate School Jozef Stefan, Jamova 39, SI-1000 Ljubljana, Slovenia; 3 Department of Biology, Institute of Molecular Systems Biology, ETH Zürich Otto-Stern-Weg 3, 8093 Zürich, Switzerland; 4 Faculty of Chemistry and Chemical Technology, University of Ljubljana, Vecna pot 113, SI-1000 Ljubljana, Slovenia

**Keywords:** cathepsin, inflammation associated disease, cancer, osteoporosis, extracellular matrix

## Abstract

For a long time, cysteine cathepsins were considered primarily as proteases crucial for nonspecific bulk proteolysis in the endolysosomal system. However, this view has dramatically changed, and cathepsins are now considered key players in many important physiological processes, including in diseases like cancer, rheumatoid arthritis, and various inflammatory diseases. Cathepsins are emerging as important players in the extracellular space, and the paradigm is shifting from the degrading enzymes to the enzymes that can also specifically modify extracellular proteins. In pathological conditions, the activity of cathepsins is often dysregulated, resulting in their overexpression and secretion into the extracellular space. This is typically observed in cancer and inflammation, and cathepsins are therefore considered valuable diagnostic and therapeutic targets. In particular, the investigation of limited proteolysis by cathepsins in the extracellular space is opening numerous possibilities for future break-through discoveries. In this review, we highlight the most important findings that establish cysteine cathepsins as important players in the extracellular space and discuss their roles that reach beyond processing and degradation of extracellular matrix (ECM) components. In addition, we discuss the recent developments in cathepsin research and the new possibilities that are opening in translational medicine.

## 1. Introduction

Cysteine cathepsins are an important group of proteases that regulate numerous physiological processes and are normally found in high concentrations in endosomes and lysosomes where they are crucial for protein breakdown and major histocompatibility complex (MHC) class II-mediated immune responses [1,2]. There are 11 cathepsins encoded in the human genome (B, C, F, H, K, L, O, S, V, W, and X) [3], and different studies have shown that a number of them have largely overlapping specificities [4,5,6,7]. Nevertheless, there are several examples of specific proteolytic functions of cathepsins demonstrating that their roles are not limited to the endolysosomal system. Cathepsins have thus been found in the cytoplasm, cell nucleus, and the extracellular space [8,9], and their extra-lysosomal localization and activity is frequently associated with ongoing pathological processes [10]. Moreover, high cathepsin activities, especially in extracellular spaces, are a hallmark of inflammation that often accompanies different diseases including cancer, arthritis, cardiovascular disease, and bone and joint disorders as a consequence of dysregulated localization, activation or transcription, as well as inhibitor imbalance [10,11,12].

Extracellular cathepsins have been shown to participate in extracellular matrix remodeling by degrading abundant structural components of the extracellular matrix (ECM) (e.g., collagen or elastin), but their extracellular functions go beyond simple proteolysis [13,14,15]. Accordingly, cathepsins have been found to be involved in the processing of cytokines and chemokines, thereby representing an important bridge between inflammation and diseases like cancer and psoriasis [16,17,18,19,20]. Moreover, other more specific functions of cathepsins were reported recently, and cathepsins were thus found to shed a group of extracellular receptors and cell adhesion molecules, demonstrating that their limited extracellular proteolysis could not only directly impact the cell surface but also influence intracellular signaling pathways (e.g., kinase receptor signaling), thereby contributing to the disease progression [21]. In addition, cathepsin S was identified as the critical sheddase of the membrane-anchored chemokine fractalkine, thereby critically contributing to the neuropathic pain [22,23].

With the emerging novel roles of cathepsins, new possibilities are opening for the development of diagnostic and therapeutic tools that will further improve our understanding of their extracellular roles and support of translational medicine. In this review, we focus on cysteine cathepsins and their roles in limited proteolysis in the extracellular space. We describe their roles in pathologies, highlight their most important disease-specific extracellular substrates, and discuss how the new findings can translate into improved diagnostic and therapeutic tools. We also discuss the future perspectives of cathepsin research that will benefit from the emerging systems biology approaches.

## 2. Cysteine Cathepsins: Structure, Function, and Regulation

**Figure 1:**
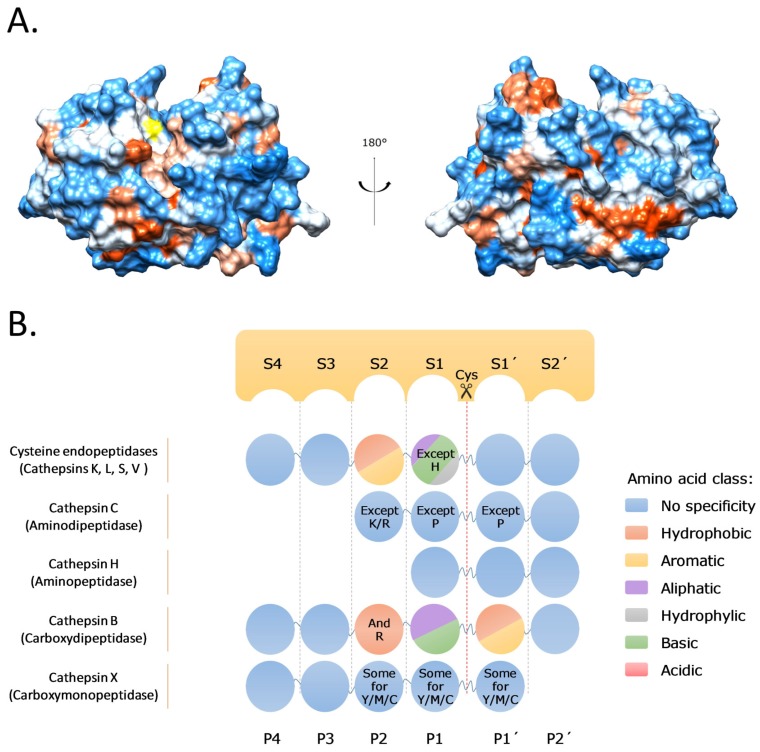
Cysteine cathepsin structures and specificities. (**A**) Crystal structure of the two-chain form of cathepsin L (PDB 1icf [33]) in standard orientation colored according to surface hydrophobicity (red: most hydrophobic, blue: most hydrophilic). Active site Cys25 is colored in yellow. (**B**) Substrate specificity of different cysteine cathepsins relative to the cleavage site, which is between the P1 and P1’ residues. S4–S1 and S1'–S2' represent the substrate binding sites into which the corresponding substrate residues P4–P2’ bind, with P4–P1 designating the substrate residue N-terminals of the cleavage site and P1’–P2’ designating residue C-terminals of the cleavage site, respectively. Amino acid residues of the substrate, which bind to their designated sites, are shown with colored circles, with each color representing a different amino acid class required for the binding of the substrate to the active site.

Cysteine cathepsins belong to the papain family of cysteine proteases sharing the typical papain-like fold, which is composed of two domains (L—left domain, R—right domain) that form the active protease with the catalytic Cys-His ion pair located in the active site cleft on opposite sides (Cys25-His159; papain numbering) [24] (Figure 1A). In general, cysteine cathepsins are monomers with a MW in the 20–35 kDa range, with variations being the consequence of different posttranslational modifications (e.g., glycosylation). The only exception is the tetrameric cathepsin C with a MW of 200 kDa. Several cysteine cathepsins, including cathepsins B, C, F, H, L, O, and X, are ubiquitously expressed in human tissues and cells, whereas cathepsins K, S, V, and W have more specific localization due to their more specific functions (Table 1). Cathepsin K is expressed in osteoclasts and synovial fibroblasts, cathepsin S is expressed predominantly in immune cells, cathepsin V in thymus and testes, and cathepsin W in CD 8+ lymphocytes and natural killer (NK) cells [1,3,12]. All cathepsins are synthesized as proenzymes, and, after activation, their activity is kept under tight control by pH, compartmentalization, and by their endogenous protein inhibitors stefins, cystatins, kininogens, thyropins, and serpins, which are important for the fine-tuning of their proteolytic activity [25,26].

The majority of cathepsins are potent endopeptidases; nevertheless, some cathepsins have exopeptidase activities due to loops and propeptide regions that limit the accessibility of the active site. Accordingly, cathepsins B and X are carboxypeptidases and cathepsins C and H are aminopeptidases, although cathepsin B can also have endopeptidase activity at a neutral pH [2]. Moreover, cathepsins are not very specific enzymes with highly similar substrate specificities and a moderate preference for cleavage after basic and hydrophobic residues. The only substrate recognition site that actually forms a defined pocket is the S2 site, which, together with the S1 and S1’ sites, seems to be the major substrate recognition site. Several studies using combinatorial peptide libraries and proteomic approaches have demonstrated a strong preference for small hydrophobic amino acid residues (Leu, Val, Ile) in the P2 position, although aromatic amino acid residues (Phe, Tyr) are also accepted (Figure 1B). However, there are a few exceptions. The first is the acceptance of Pro in the P2 position of cathepsin K, which is important for the collagenolytic activity of the latter. The second is the acceptance of Arg in the P2 position of cathepsin B [4,5,6,7,24]. The broad specificity of cathepsins is in good agreement with their roles in protein turnover and degradation, including in antigen processing. On the other hand, such broad substrate specificity combined with the high proteolytic efficiency ensures that even in unfavorable conditions, cathepsins can have major roles not only in protein degradation but also in more subtle protein processing, thereby having more signaling roles. This is supported by numerous examples where several cathepsins were found to cleave their protein substrates at the same or similar cleavage sites, although there were also examples of substrates like collagen, osteocalcin, cytokines, and chemokines, which were only cleaved by a subset of cathepsins [10,14].

For their optimal activity, cysteine cathepsins require reducing and mildly acidic conditions and, except cathepsin S, all are irreversibly inactivated at a neutral pH with cathepsin L being the most unstable [34,35,36,37]. While these findings originate mostly from in vitro assays, different components in the extracellular milieu and ECM building blocks can stabilize or alter cathepsin activity in the extracellular milieu. A good example is the glycosaminoglycans (GAGs) that can stabilize cathepsins at a neutral pH [38]. Moreover, experiments have shown that GAGs and other negatively charged surfaces enable autocatalytic activation of cathepsins even at neutral pH and possibly contribute to the cathepsin activity in the extracellular space [39]. Nevertheless, effects can be substantially different. Accordingly, the collagenolytic activity of cathepsin K is reduced in case of dermatan sulfate, heparan sulfate, and heparin, but GAGs like keratan and chondroitin sulfates can potentiate it [40]. Another good example is chondroitin sulfate, which inhibits the elastolytic activity of cathepsins V, K, and S [41]. In addition, very potent regulators of cysteine cathepsins are their endogenous inhibitors: cystatins (stefins, cystatins, and kininogens), thyropins, and serpins. They inhibit most of the cathepsins with very high affinities in the nM to pM range. While stefins are essentially cytosolic, the others are primarily extracellular, and their main function is to block the cathepsins escaped into the extracellular milieu, thereby acting as emergency-type inhibitors [1,26,42].

## 3. Extracellular Cysteine Cathepsin Origins

There is long-lasting evidence that cysteine cathepsins can be present in the extracellular milieu. Under physiological conditions, they are commonly involved in the processes of wound healing, bone remodeling, and prohormone activation [9,43]; however, their extracellular localization is far more common in different pathological conditions [10,12,44]. Prolonged extracellular cathepsin activity upon their secretion is unusual since they have optimal stability under acidic conditions. Nevertheless, the loss of activity at neutral pH can at least partly be delayed by secretion in zymogen form in which they remain relatively stable until their activation [45,46]. Apart from that, cysteine cathepsins can be secreted in an active form and their concentration is sufficient for ECM degradation [47]. An important factor for their secretion is also the type of cell. High levels of cathepsins are most commonly secreted by different immune cells [48], which is in agreement with the fact that cathepsins are elevated in inflammation [49]. In addition, keratinocytes, osteoclasts, smooth muscle cells, and thyroid cells also secrete cathepsins [10]. Nevertheless, the concentration is much lower than in the case of immune cells, where it can reach up to 100 nM in the macrophage secretome [21].

**Figure 2:**
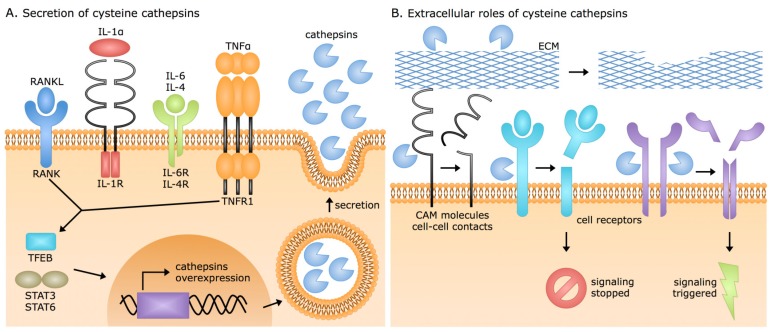
Cysteine cathepsin secretion and their extracellular roles. (**A**) Secretion of extracellular cathepsin is often tightly connected with their overexpression and can be triggered by diverse cell signaling pathways. Overexpressed cysteine cathepsins are usually secreted with vesicular exocytosis. (**B**) In the extracellular milieu, cysteine cathepsins cleave different targets. Cleavages of cell adhesion molecules (CAM), cell-cell contacts, and proteins of ECM mainly influence cell adhesion and migration. Additionally, proteolytic products of these cleavages can act as signaling molecules and have an impact on cell growth, invasion, and angiogenesis. Other main target of cysteine cathepsins are cell receptors, and their cleavage can result in either constantly triggered signaling, in the case of partial trimming of the receptor, or inhibited signaling, in the case of a complete removal of the extracellular domain. CAM, cell adhesion molecules; ECM, extracellular matrix; IL, interleukin; RANK, receptor activator of NF-κΒ; RANKL, receptor activator of NF-κΒ ligand; STAT, signal transducer and activator of transcription; TFEB, transcription factor EB; TNFα, tumor necrosis factor alpha; TNFR1, tumor necrosis factor receptor 1.

Diverse cellular mechanisms and pathways were shown to be involved in the secretion of cysteine cathepsins (Figure 2A), which is often accompanied by acidification of the extracellular milieu. Experiments performed with macrophages showed that acidification can be achieved either by vacuolar-type H^+^ ATPase, which undergoes activation by protein kinase C or serotonin [50,51,52] or by Na^+^/H^+^ exchanger I, activated following the binding of immunoglobulin E to high-affinity immunoglobulin-ε receptor [53]. Acidification is especially pronounced in the tumor microenvironment, where tumor-associated immune cells secrete large amounts of cathepsins, and their extracellular presence is connected with more aggressive cancers and inflammation [54]. Apart from cancer, acidification was also observed to be present in advanced osteoarthritis [55] and in atherosclerotic plaques [56]. Usually, secretion of cysteine cathepsins is linked with their overexpression, a common consequence of activation of transcription factor EB [57], or signal transducer and activator of transcription (STAT) signaling pathways by activation of STAT3 or STAT6 [58,59]. Elevated expression can also be the result of extracellular stimuli provided by different cytokines and interleukins [60,61]. The main examples of cytokine-triggered overexpression and secretion are cathepsins S and K. Cathepsin S is overexpressed and secreted in its active form by human chondrocytes upon stimulation from pro-inflammatory cytokines interleukin 1α (IL-1α) and tumor necrosis factor α (TNF α) [60], while regulation of cathepsin K expression is controlled by RANKL (receptor activator of NF-κB ligand; NF-κB, nuclear factor kappa-light-chain-enhancer of activated B cells) [62]. Another factor that can act as an initiator of the positive feedback loops that drive the cathepsin secretion are proteolytic products of ECM degradation [63], whereas elevation of intracellular levels of Ca^2+^, which triggers the fusion of lysosomes with the plasma membrane, enables secretion of cathepsins by vesicular exocytosis [64,65]. In addition, secretion of cathepsins may even result from increased concentrations of reactive oxidative species, which may lead to the permeabilization of the lysosomal membrane and the release of lysosomal proteases into the cytoplasm and further into the extracellular milieu [66,67].

Once secreted, cathepsins can either remain bound to the plasma membrane or interact with molecules from the extracellular milieu [15]. In particular, the former may be of special importance as such membrane association may also protect the cathepsins against inactivation in otherwise unfavorable conditions of the extracellular milieu. Cathepsin S was thus shown to associate with the plasma membrane and co-localizes with α_v_β_3_ integrin on the surface of smooth muscle cells present in the vasculature [68], but its exact binding partners remain unknown. Another cathepsin that translocates to the plasma membrane is cathepsin X, which acts as an activator of β_2_ integrins that are crucial for cell adhesion of dendritic cells and lymphocytes during their maturation [69,70,71]. Translocation of cysteine cathepsins occurs also in pathological conditions as shown for cathepsin B, which localizes to the cell membrane in cancer cells [72] either by binding to annexin II tetramers [73] or by association to the caveolae site [74]. Moreover, its membrane-bound localization was correlated with shortened survival in the case of colorectal cancer [75,76].

## 4. ECM Proteolysis and the Cathepsins

The ECM that surrounds the cells is composed of the proteins these cells secrete [77]. The main components of ECM are structural proteins, like collagen, elastin, fibronectin and laminins, non-structural matricellular proteins, polysaccharides, mainly glycosaminoglycans and hyaluronan, and proteoglycans [78,79]. The structure of the ECM is dynamic and depends on the equilibrium between synthesis and degradation [80,81]. Modifications of the ECM are achieved through both enzymatic and non-enzymatic processes, which can influence the stability and function of the ECM [82]. Among these processes, proteolytic processing is one of the most important mechanisms involved in the regulation of the ECM. It is therefore not surprising that a vast number of proteases were found to be involved, with major players being various metalloproteases such as a disintegrin and metalloprotease (ADAMs), matrix metalloprotease (MMPs), meprins and bone morphogenetic protein (BMP) /tolloid metalloproteases [83] and various cysteine proteases [15].

Cysteine cathepsins are the main cysteine proteases participating in the reorganization of ECM and are associated with non-specific degradation of abundant ECM proteins, which can take place both extracellularly and intracellularly following endocytosis [82]. Proteolysis of ECM proteins occurs under normal conditions, as in the case of collagen degradation in bone resorption or elastin degradation in the vascular system, as well as under pathological conditions, since ECM degradation is an influential factor in many diseases including cancer, cardiovascular diseases, and arthritis [14,15,84]. In particular, extracellular proteolysis seems to be of crucial importance, since the extracellular cathepsins can cleave a plethora of structural and functional proteins, thereby affecting not only the structural aspects of ECM but also the associated signaling pathways (Figure 2B). While non-specific and specific proteolysis by cysteine cathepsins are important for ECM remodeling in disease, one has to keep in mind that extracellular substrates of cysteine cathepsins were mostly identified in in vitro studies. Nevertheless, much evidence for their physiological significance and substrates confirming their involvement in disease development and progression has emerged just in the last decade (Table 2), and the next sections will provide a detailed overview of the extracellular roles of cathepsins in different pathologies.

### 4.1. Cathepsins in Cancer

Cysteine cathepsins were historically first linked to extracellular proteolysis in cancer, as first demonstrated for cathepsin B almost 40 years ago [106]. However, despite the use of various in vivo and in vitro cancer progression models, the exact roles of individual cysteine cathepsins are not completely understood [107,108,109]. A comprehensive understanding of the functions of cathepsins B, C, H, K, L, S, and X in the extracellular milieu has proved complicated because of their broad substrate specificity [1], their endogenous inhibitors [110], compensatory effects [111,112], effects that are not associated with their proteolytic function [113], their ability to act as tumor suppressors [114], and the various different cell types that comprise many tumors [115].

Cathepsins are released into the tumor microenvironment by different cells including tumor cells, endothelial cells, tumor-associated macrophages (TAM), myoepithelial cells, fibroblasts, and other cells, which infiltrate the tumor site [48,54]. Among these, TAMs are considered to release the largest amount of cathepsins primarily due to stimulation by the IL-4, IL-6, and IL-10 cytokines in the tumor microenvironment [10,48]. Interestingly, the source and role of secreted cathepsins responsible for cancer progression are not universal among different types of cancer, as evident primarily from different mouse *in vivo* cancer models. In the RIP1-Tag2 model of the pancreatic neuroendocrine cancer, cathepsins B, H, and S, derived from the TAMs, had a predominant, if not exclusive, effect on cancer progression through the reduction of tumor burden, increase in apoptosis, and decrease of angiogenesis [48]. On the other hand, cathepsin L-mediated cancer progression was largely due to its secretion from cancer cells, whereas the effect of cathepsin X was due to its release from both sources. Moreover, cathepsin X was found to partially compensate for the loss of cathepsins B and S, as revealed by simultaneous deletion of the two cathepsin genes. In addition, simultaneous deletion of cathepsins B and S revealed additive effects in early stages, but at late stages, several differences were restored to the wild-type level, although the mechanisms are not known [111,113]. Interestingly, expression of cathepsin C was also increased in RIP1-Tag2 and MMTV-PyMT mammary gland models of cancer but had no functional role in their progression [109,116]. Cathepsin S was also found to have a major role in a syngeneic colorectal carcinoma murine model, where its release from cancer cells, endothelial cells, and TAMs was found to be responsible for the progression of cancer through the promotion of neovascularization and tumor growth [117]. An analogous effect was also observed *in vivo* in the MMTV-PyMT mammary gland cancer model, where cathepsin B, originating from both tumor cells and macrophages, was suggested to have a major role in the tumor progression and metastasis spread [107,118], which could have been partially compensated by cathepsin X [112]. However, using an orthotopic transplantation of primary mouse PyMT cancer cells overexpressing cathepsin B showed that the enzyme expressed and secreted from tumor, but not stromal, cells increased invasiveness into adjacent tissues by excessive extracellular matrix degradation [119]. Another *in vivo* mouse model, where the roles of individual cathepsins were systematically investigated, was the K14-HPV16 model of squamous cell carcinoma. In this model, cathepsin C, but not cathepsin B, released from stromal cells was shown to be responsible for cancer development and angiogenesis. Moreover, cathepsin L was found to have a tumor suppressor role in this model, further showing the complexity of cathepsins involvement in tumorigenesis [109].

There are also numerous cellular studies available where secretion of cathepsins was shown to be an important factor in changing cellular properties possibly leading to tumor progression, although these studies were mostly focused on tumor cell lines but not on macrophages or related immune cells. Among the cathepsins, cathepsin B was most often associated with changes of cellular properties, although there were also numerous reports about cathepsins L, X, and K. Cathepsins B and X were thus shown to be important in the epithelial to mesenchymal transition in two breast cancer cell lines, MCF7 and MDA-MB-231, which express low and high amounts of cathepsins, respectively. Upregulation of cathepsins B and X in MCF7 cells decreased E-cadherin, a marker for epithelial cells, which was mainly attributed to cathepsin B [120]. Similar observations involving cathepsin X were also reported in hepatocellular cancer where its overexpression increased invasiveness and motility in a matrix-coated transwell model and a wound healing assay [121], although the enzyme seems to be primarily expressed in the immune cells [70]. Release of cathepsins B and L was recently reported in different melanoma cell lines, where Abl/Arg nonreceptor tyrosine kinases were shown to play an important role in cathepsin B and L expression and their release from the cells [122]. Tumor progression and increased cell invasiveness following cathepsin B release are also characteristic for pancreatic ductal adenocarcinoma [123], esophageal adenocarcinoma [124], and glioma, where cathepsins K and X were also found to be involved [125,126,127]. Recently, a mechanism of cathepsin B release followed by enhanced lung cancer cell migration was proposed [128], indicating a possible role in the progression of lung cancer. However, the role of cathepsins in cancer is not only extracellular but also has an important intracellular component [48,129]. An example of this is the leakage of cathepsin B to the cytoplasm following chemotherapy-induced lysosomal membrane permeabilization in myeloid-derived suppressor cells (MDSC). Upon release, cathepsin B was found to interact with NLRP3, leading to a non-proteolytic activation of the inflammasome and pro-IL-1β processing. The released IL-1β enhanced IL-17 production in CD 4^+^ T cells, which resulted in angiogenesis and tumor relapse [130].

Unfortunately, there are almost no in vivo studies on the role of cysteine cathepsin inhibitors that could shed some more light on the possible imbalance between the endogenous inhibitors and the target proteases as one of the reasons for tumor progression, as suggested some time ago [54]. The first study revealed that genetic ablation of cystatin C, major extracellular cathepsin inhibitor, in the pancreatic neurocrine tumor model (Rip-Tag2) resulted in an increased size of islet cell carcinomas and angiogenic islets, which was linked to deregulated cathepsin S activity leading to increased endostatin generation [91]. The other two studies available included genetic ablation of stefin B, the major intracellular cathepsin inhibitor [131], and of cystatin C [132], in the mammary gland PyMT mouse model. However, contrary to all expectation, tumors were smaller in both inhibitor knock-out models despite the increased cathepsin activity in the tumors. While in the case of stefin B this was linked to increased sensitivity to lysosomal cathepsin-mediated cell death and oxidative stress, the potential link in case of cystatin C were the 14-3-3 proteins, but the evidence is not entirely conclusive. This further supports the idea of differential and context-dependent roles of cathepsins and their inhibitors in cancer.

Nevertheless, it seems that the major role of cathepsins in cancer is in extracellular matrix degradation [87], shedding of receptors and adhesion molecules [21,85], activation of cytokines and growth factors [48], and cleavage of proteins forming cell-cell junctions [133]. In addition, cathepsins were also suggested to be involved in the activation of other tumor-associated proteases, although the evidence is primarily based on in vitro studies. An example is cathepsin B, which was suggested to be involved in the activation of urokinase-type plasminogen activator (uPA), which influences uPA/uPAR signaling, thereby possibly affecting cell migration, whereas cathepsin L was suggested to be involved in the activation of MMP1 and MMP3 [13,54].

Despite many phenotypic changes following cathepsin ablation or inhibition in in vivo and in vitro tumor models, the exact roles, besides the degradation of extracellular matrix, are less-well known. This is supported by a recent proteomic study where individual genetic ablation of cathepsins B, H, L, S, and X in the Rip-Tag2 pancreatic tumor model was shown to have a predominant effect on the degradation of the extracellular matrix, with very few limited proteolysis events detected [87]. Nevertheless, limited proteolysis of E-cadherin, an important adhesion molecule and marker for epithelial phenotype, by cathepsins B, L, and S was shown to potentially drive invasiveness of cancer cells in the same RIP1-Tag2 model [116]. There are several other substrates of individual cathepsins identified in different cancer models, but their importance for cancer progression is unclear. Among these targets is CD18, which was found to be cleaved by cathepsin B released from adhering leukocytes following physiological levels of shear stress [134] and may have a role in leukocyte recruitment in angiogenic vessels [85]. Another set of substrates of extracellular cathepsins associated with angiogenesis are basement membrane proteins, which are cleaved during basement membrane degradation, in mother vessels formation, following vascular endothelial growth factor A (VEGF-A) stimulation [135]. One of these is nidogen-1, which was found to be degraded by cathepsin S in patients with non-small cell lung cancer [136]. In addition, cathepsins B, L, and S were reported to cleave laminin, with cathepsin S generating a fragment with pro-angiogenic effects [91]. Cathepsins B, L, and S can also cleave fibronectin, the results of which are still poorly understood [15,88]. Another such substrate is tenascin-C, which was found to be cleaved by cathepsin B, resulting in a pro-angiogenic effect in glioma [86]. An important cathepsin substrate also seems to be collagen XVIII, which can serve as a source of endostatin that can be generated by cathepsins L and S, thereby affecting angiogenesis [137,138]. Finally, cathepsin K was demonstrated to cleave periostin, which may be linked to breast cancer bone metastasis. The decrease of C-terminal intact periostin was namely shown to be a marker for osteolytic lesions in breast cancer bone metastasis and could be used to detect bone relapse in patients [89]. Cathepsin K cleavage of osteonectin was also implicated in experimental prostate to bone tumor metastasis, where both the protease and substrate expression levels were higher. Additionally, high levels of released inflammatory cytokines suggested that cathepsin K and/or osteonectin may regulate their release [90]. Some additional cathepsin ECM substrates linked to cancer have also been described elsewhere [15,82]. A more detailed overview on the roles of cysteine cathepsins in cancer development and progression can be found elsewhere [48].

### 4.2. Cysteine Cathepsins and Tissue Remodeling

The role of cathepsins in bone and cartilage processing is extremely well described, with cathepsin K being the most studied. The main source of cathepsin K in these processes are osteoclasts. Cathepsin K is one of the few proteases capable of cleaving the collagen triple helix in the polyproline region, and its activity differs from metalloproteases. It has multiple cleavage sites in the polyproline region of both collagens I and II [102,139] and can also cleave collagen in the telopeptide region [101]. Collagenolytic activity of cathepsin K is strong in the acidic environment at the site where osteoclasts attach to the bone surface and is crucial for normal bone homeostasis [14]. It has been shown that inhibition of cathepsin K can result in an altered structure of the bone, leading to changed crystallinity and crystal structure [140]. Another factor that influences cathepsin K activity is the presence of GAGs, since its collagenolytic activity is completely dependent on the formation of an oligomeric complex between cathepsin K and GAGs [141,142]. Aging-associated changes of collagen fibers were also suggested to influence the collagenolytic activity of cathepsin K. Accumulation of advanced glycation end-products (AGEs) and mineralization were thus shown to reduce cathepsin K collagenolytic activity, while removal of GAGs completely blocked it [143]. Pathological collagen degradation is connected with cathepsin K overexpression and unbalanced osteoclast/osteoblast activation, leading to collagen I degradation and bone loss as seen in osteoporosis [144]. While cathepsin K is the most important cathepsin in bone and cartilage remodeling, one must keep in mind that in vitro studies have identified cathepsins B, L, H, and S as potentially being involved in bone remodeling since they can cleave osteocalcin [103], a biomarker of bone degradation in osteoporosis [145]. 

Other pathological conditions with similar causes are osteoarthritis and rheumatoid arthritis, where cathepsin K degrades collagen II from the N-terminus, which leads to cartilage erosion [99,102]. Moreover, the low pH, as observed in advanced osteoarthritis, favors cathepsin K as a major collagenase in these diseases [55]. In addition, cathepsin K can cleave the proteoglycan aggrecan, which is also cleaved by cathepsins B, L, and S [98,100], and ECM bone protein osteonectin [104], further supporting its crucial role in osteoarthritis. However, in vivo studies in mouse models showed that disease progression was substantially diminished in cathepsin S-deficient animals in collagen-induced arthritis and in cathepsin L-deficient animals in antigen-induced arthritis [29], suggesting that other cathepsins are also involved in the arthritis progression. Moreover, substantial levels of cathepsins B, L, and S were identified in synovial fluids of patients with rheumatoid arthritis and osteoarthritis, with higher levels detected in rheumatoid arthritis patients, suggesting their involvement in inflammation and cartilage destruction [146,147,148,149,150]. However, despite high serum levels of cathepsins S and L in rheumatoid arthritis patients, they did not correlate with the severity of the disease, arguing against their use as disease biomarkers [151]. Anyhow, substantial differences in the serum levels of these two cathepsins were observed between the rheumatoid arthritis patients with and without autoantibodies, suggesting specific roles of cathepsins S and L in disease development and progression in seropositive patients [150].

Cysteine cathepsin-mediated ECM remodeling is also important in the cardiovascular system, where cathepsins K, S, and V can cleave elastin, with cathepsin V displaying the highest elastolytic activity [84]. This is a result of two unique exosites present in its structure, which stabilizes the elastin-cathepsin V complex [92]. Since elastin is responsible for tissue stability against stretching forces, its fragmentation decreases the elasticity of the blood vessels and can lead to their rupture [152]. Excessive elastin degradation was observed in cardiovascular diseases, atherosclerosis and abdominal aortic aneurism, kidney diseases, and during aging [15,84], as upon reaching adulthood, elastin is not synthesized de novo anymore, and consequently its degradation is irreversible [153]. Besides cathepsin S, cathepsin K and V also exhibit elastinolytic activity important in cardiovascular pathology, and their ablation in mice showed decreased signs of atherosclerotic pathology [154,155,156]. Elastin degradation by cysteine cathepsins occurs mainly extracellularly, with only one-third of elastin being degraded intracellularly [41]. However, not all functions of extracellular cysteine cathepsins in the cardiovascular system are harmful. A recent study has shown that cathepsin K has a pivotal role in cardiac remodeling following myocardial infarction, since sufficient collagen degradation is needed to reduce cardiac fibrosis [93]. However, processing of membrane-bound chemokine fractalkine (CX_3_CL1 or (C-X3-C motif) ligand 1) by cathepsin S homes additional inflammatory cells to the site of atherogenesis, thereby sustaining the inflammation and progressing the pathology [157]. The ECM degradation capability of cathepsin B is also considered to be a marker of the disease, whereas cathepsin X has a role in homing the inflammatory cells, especially T-cells, to the atheroma site [158].

Cysteine cathepsins B and L have an important role in neural tissue remodeling, especially in axon growth, but exact mechanisms remain mostly unknown [159,160]. Nevertheless, both can cleave perlecan, and this cleavage generates a C-terminal fragment with neuroprotective roles [161]. Another role of cathepsin B is the degradation of chondroitin sulfate proteoglycans (CSPGs) [160], which have inhibitory effects on axon growth and regeneration transmitted by receptor protein tyrosine phosphatase σ (RPTKσ) [162]. A recent study found that regulation of cathepsin B expression and secretion by intracellular sigma peptide (ISP)-modified RPTKσ signaling can possibly lead to axon overgrowth in a CSPG-rich environment [160]. Another study also identified cathepsin B as a myokine that is systemically secreted during running and can cross the blood-brain barrier. This running-induced, systemically secreted cathepsin B induced neurogenesis, improved spatial memory, and also influenced plasticity, cell survival, differentiation, and neuronal migration [163]. In addition, in vitro experiments have also identified extracellular cathepsin L as a potential stimulus of axonal growth [159].

Cysteine cathepsins also participate in lung pathologies [164]. In silicosis, cysteine cathepsins B, H, K, L, and S were found in large excess compared to their inhibitors in the patient's bronchoalveolar lavage fluid (BALF) and are likely involved in the breakdown and remodeling of the ECM [165]. Recently, cathepsin K overexpression and release was also found in lymphangioleiomyomatosis (LAM), a rare nodule-forming disease. Interestingly, cathepsin K was overexpressed and released from fibroblasts associated with LAM cells similar to tumor cell–stroma interaction and could be the driving factor behind matrix degradation and cytokine processing [166]. In the lung, cysteine cathepsin activity is involved in tuberculosis where cathepsin K is one of the collagenolytic proteases that degrade the ECM and cause the formation of lung cavities [105,167]. On the other hand, cathepsin K overexpression and extracellular activity warranted protection against bleomycin-induced pulmonary fibrosis. Additionally, cathepsin K knock-out mice deposited more extracellular matrix and had decreased collagenolytic activity, which points to an important role of cathepsin K in lung collagenolytic activity [168,169]. The same model also provided evidence for cathepsin B overexpression and presence in BALF [170]. Furthermore, cathepsin S was shown to cleave decorin and produce a fragment, which can be robustly detected in the serum of fibrosis and cancer patients [94]. Finally, pharmacological inhibition of cathepsin S was shown to substantially reduce a cystic fibrosis-like disease in a mouse model, possibly via a link to protease-activated receptor 2 (PAR2) [171], which was previously reported to be a cathepsin S substrate [172].

More examples of cathepsin-mediated tissue remodeling are scattered throughout tissue types. In the case of pre-adipocyte cells, cleavage of fibronectin by cathepsin S was suggested as a possible mechanism for their differentiation [173]. Recent in silico and in vitro studies reported that cathepsins K, L, and S could cleave fibrinogen and fibrin, resulting in fragments that differ from the ones produced by plasmin, which opened the door to further investigate their roles in vascular homeostasis, especially in coagulation-associated diseases [174,175]. Moreover, cathepsin K was recently demonstrated to have an essential role in skeletal muscle remodeling, dysfunction, and fibrosis following injury [176]. The roles of cysteine cathepsins in tissue remodeling are diverse and can be both detrimental and beneficial; therefore, cysteine cathepsins are emerging as possible targets in therapeutic strategies.

### 4.3. Cysteine Cathepsins in Inflammation

Cysteine cathepsin secretion often accompanies different inflammation-driven pathologies where they are released from recruited immune cells or aberrantly expressed or processed in the inflamed tissue. Because of this general mechanism, there are numerous organ systems or tissues where cathepsins can be detected extracellularly during inflammation, and their extracellular localization has been implicated in different aspects of the immune cell physiology [10]. Extracellular cathepsins, in particular cathepsins L, S, and K, were shown to process and activate the glutamate-leucin-arginin motif (ELR) and inactivate the non-ELR CXC (N-terminal Cys-X-Cys motif) chemokines, thereby regulating chemotaxis and angiogenesis [16]. In the case of cathepsin S, its extracellular localization can influence macrophage and monocyte migration through the basal membrane [154]. The arginin-glycin-aspartate (RGD) motif in procathepsin X is responsible for its binding to integrin α_v_β_3_, thus modulating the binding of cells to the ECM components [177]. In intestinal goblet cells, cathepsin K is highly expressed and released and provides an antimicrobial effect. As a result, cathepsin K-deficient mice exhibit more severe colitis and have an altered microbial community [178]. During inflammation, a crosstalk is established with the coagulation cascade where fibrin, which can be cleaved by cathepsins K, L, and S in vitro, plays an important role [179].

Moreover, it seems there is also a connection between the nervous and immune systems, where cathepsins were suggested to play a role [180]. At this neuroimmune interface, cathepsin S has been shown to cleave PAR2 in a different pattern as previously described for serine proteases, inducing hyperalgesia [96]. Additionally, cathepsin S cleavage of CX_3_CL1 from neurons results in microglial stimulation, which is critical in chronic pain maintenance [181]. Furthermore, microglia cells were shown to secrete cathepsin B, S, and X, which are considered important in inflammation-induced neurodegeneration [182].

Cysteine cathepsins also play a role in both acute and chronic phases of kidney disease, albeit there are currently more known intracellular functions compared to extracellular ones [183]. Nevertheless, cathepsin L-mediated heparanase activation was shown to play a role in the pathogenesis of diabetic nephropathy, causing proteinuria and renal damage [97]. Finally, cathepsin S elastolytic activity was also implicated in the occurrence of calcifications during the pathogenesis of chronic kidney disease [184].

## 5. Extracellular Cathepsins and Their Translation into Clinical Applications

**Figure 3:**
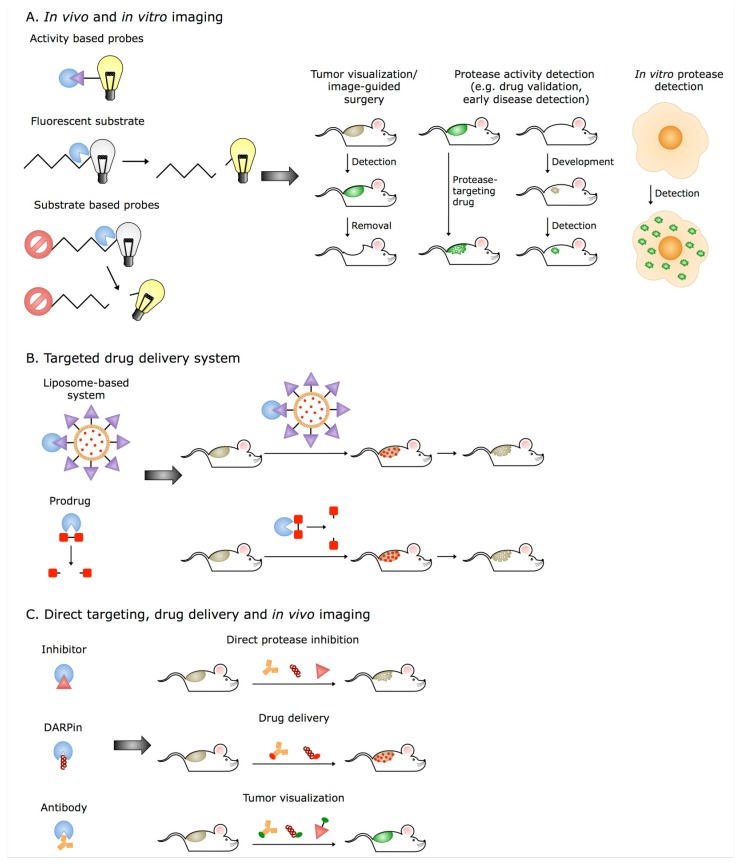
Extracellular cathepsins as diagnostic targets, prodrug activators, and targets for targeted drug delivery. The high levels of cathepsins in the ECM can be utilized in different imaging and targeting techniques. (**A**) Fluorescent substrates, substrate-based probes, and activity-based probes are the most commonly used tools for in vivo and in vitro imaging. (**B**) Extracellular cathepsins can be also used for targeted drug delivery and for prodrug activation. (**C**) Since many cathepsins are also overexpressed and active in different cancers, they can be targeted directly using their inhibitors, designed ankyrin repeat proteins (DARPins), or antibodies. These molecules can also be conjugated and, as such, used for drug delivery and tumor visualization.

Elevated activity of cysteine cathepsins in the extracellular space is now widely recognized as an important hallmark of developing or ongoing disease. Therefore, cathepsins are getting increasing attention in the development of novel therapeutic and diagnostic tools (Figure 3). In particular, therapeutic inhibition of cathepsins was the driving force in the field since the discovery that cathepsin K has a crucial role in bone resorption and thus in osteoporosis [37,104,185,186], and that cathepsin S is the key enzyme in the MHC II-mediated immune response [29,31]. A number of small-molecule inhibitors of cathepsins were developed, and despite several inhibitors of cathepsin K and S showing good initial results for treatment of osteoporosis, aortic aneurysm, arthritis, and neuropathic pain, none has entered clinical use so far, and very few are in clinical trials at the moment [10,84]. There are several reasons for this, with perhaps the best-known being the on-target toxicity revealed in the case of cathepsin K inhibitors, which became evident after long-term treatment. This was demonstrated in the case of Odanacatib (Merck), a non-basic nitrile, which was very successful in preclinical stages and even successfully concluded phase III clinical trials, but prolonged investigations of the stroke-related side effects lead to its discontinuation [84,187,188]. However, the problem was already at least partially raised with Balicatib (Novartis), the first cathepsin K inhibitors that entered clinical trials for osteoporosis treatment and was discontinued after Phase II [188]. In order to overcome the problem, research went in the direction of exosite inhibitors of cathepsin K that would only block collagen degradation, whereas cytokine processing, such as that of TGF-β, which is active site-driven, would be unchanged. A good progress in this area was demonstrated with tanshinones, a group of so-called ectosteric inhibitors targeting the cathepsin K exosite originating from plants, which already showed good results in preclinical in vivo studies [189,190]. Currently, a lot of hope is also in the new inhibitors of cathepsin S for neuropathic pain treatment, but the outcome of the clinical studies remains to be seen [10]. Another strategy is the use of inhibitory antibodies, which demonstrated good potential for cathepsin S inhibition, resulting in reduced tumor growth and improved chemotherapeutic efficiency [191,192], and further developments are expected to be seen in this area as well.

However, the high extracellular cathepsin levels secreted from the immune cells in various inflammation-associated diseases including many cancers opened the door for in vivo diagnostic applications. Different activity-based probes (ABPs) with fluorescent tags or internally quenched substrates that start to emit the reporter signals after cathepsin cleavage were used to detect pathologic cathepsin activity [193]. There are several examples where probes were used to detect inflammation [194], visualization of cancer cells [195], and lung fibrosis [196]. Good results were also achieved with fluorogenic substrates in preclinical imaging of cathepsins, especially with the application of the reverse-design principle where medicinal chemistry-optimized small molecule inhibitors were converted to fluorescent activatable substrates [197]. A cathepsin S-selective lipidated fluorescent substrate based on this principle was successfully used for in vivo imaging of mammary gland mouse tumors [198]. Another set of tools that can be used for labeling cathepsins are designed ankyrin repeat proteins (DARPins). A cathepsin B-selective DARPin with high affinity was successfully used for in vivo imaging in two mouse models of mammary gland cancer [199]. While selective tools perform well at the preclinical level, pan-cathepsin probes were much more successful in image-guided surgery applications to visualize the tumor tissue and thus increase the chances of its complete removal [200], leading to the first compounds being evaluated in clinical trials [10].

Concepts for targeted drug delivery can also largely benefit from the elevated activity of cysteine cathepsins in disease. First, cathepsins can be used as drug activators. Accordingly, several drugs are synthesized as prodrugs or antibody-drug-conjugates (ADCs) and become active only after cathepsin cleavage. This concept has been successfully used in oncology with a good example being ADCETRIS®, which is already clinically approved [10], while several other prodrugs are at different stages of development [201,202]. Second, targeting extracellular or membrane-associated cathepsins also emerged as a promising drug delivery strategy. The power of this concept was demonstrated when cancer cell membrane-associated cathepsin B was targeted by liposomes with a selective cathepsin B inhibitor as a targeting moiety, and the system demonstrated improved selectivity and targeting efficiency [203]. However, while the majority of research has been focused on how to exploit the extracellular presence of cysteine cathepsins for medical applications, the extracellular substrate pool itself represents a major source of potential future therapeutic targets, targeting moieties, and biomarkers that will likely result in the development of new diagnostic and therapeutic strategies with major potential for future clinical applications [204].

## 6. Concluding Remarks and Future Perspectives

Recent findings on the roles of cysteine cathepsins in the extracellular space have substantially improved our understanding of these important proteases and of their normal and pathological proteolysis in the ECM. While cathepsins are widely recognized as important diagnostic and therapeutic targets largely for the diseases that involve ECM remodeling, their multifunctional roles pose a problem in the design of successful tools for their therapeutic targeting as demonstrated by the systematic failure of a number of cathepsin inhibitors in clinical trials. Therefore, we believe that in the future, the clinical paradigm on cysteine cathepsins will shift from targets for therapeutic intervention to diagnostic targets and targets for image-guided surgery, targets for targeted drug delivery, modifiers of the cancer cell surfaceome, and generators of cancer cell-specific fingerprints with biomarker potential, thereby leading to the development of new cathepsin-based diagnostic and therapeutic applications.

## Figures and Tables

**Table 1:** Cysteine cathepsins. Overview of cysteine cathepsins, their peptidase activities, expression, and gene names according to the HUGO Gene Nomenclature Committee.

Cysteine Cathepsins.	Gene Name	Peptidase Activity	Expression
Cathepsin B	CTSB	Carboxydipeptidase, Endopeptidase	Ubiquitous
Cathepsin C	CTSC	Aminodipeptidase	Ubiquitous
Cathepsin F	CTSF	Endopeptidase	Ubiquitous
Cathepsin H	CTSH	Aminopeptidase, Endopeptidase	Ubiquitous
Cathepsin K	CTSK	Endopeptidase	Osteoclasts [27]
Cathepsin L	CTSL	Endopeptidase	Ubiquitous
Cathepsin O	CTSO	Unknown	Ubiquitous
Cathepsin S	CTSS	Endopeptidase	Antigen-presenting cells (e.g., dendritic cells, B-cells) [28,29]
Cathepsin V	CTSV	Endopeptidase	Thymus, testis [30,31]
Cathepsin W	CTSW	Unknown	Natural killer cells, cytotoxic T cells [32]
Cathepsin Z (Cathepsin X)	CTSZ	Carboxymonopeptidase	Ubiquitous

If not otherwise stated, expression profiles are from ref. [3].

**Table 2:** Cathepsins in disease.

Disease	Cathepsins Involved	Cleaved Targets	Selected References
Angiogenesis/Leukocyte recruitment	B, K, L, S	ELR (glutamate-leucin-arginin motif) chemokines/non-ELR chemokines, CD18	[16,85]
Cancer	B, K, L, S, X	Tenascin-C, nidogen-1, fibronectin, osteonectin, laminin, periostin, collagen IV, general degradation	[86,87,88,89,90,91]
Cardiovascular and kidney diseases (e.g., atherosclerosis, abdominal aortic aneurysm, chronic kidney disease)	K, L, S, V	Elastin, CX_3_CL, heparanase, collagen I (catK: *Gly61-Lys62, Arg144-Gly145, Gln189-Gly190*)	[41,92,93]
Lung fibrosis	S	Decorin	[94]
Neuroinflammation/hyperalgesia	S	CX_3_CL1, PAR2	[95,96,97]
Osteoarthritis and rheumatoid arthritis	K, B, L, S	Collagen II (catK: *Gly61-Lys62, Arg144-Gly145, Gln189-Gly190*), aggrecan (catB: *Asn341-Phe342, Gly344-Val345,* catL: *Gly344-Val345*)	[98,99,100]
Osteoporosis	K, B, L, S, H	Collagen I (catK: *Gly61-Lys62, Arg144-Gly145, Gln189-Gly190*), osteonectin, osteocalcin (catB: *Arg44-Phe45,* catL: *Gly7-Ala8, Arg43-Arg44,* catS: *Gly7-Ala8*)	[101,102,103,104]
Tuberculosis	K	Collagen I (casK: *Gly61-Lys62, Arg144-Gly145, Gln189-Gly190*)	[105]

Diseases where cysteine cathepsins are involved in the development and progression of the pathology together with their extracellular substrates. Known cleavage sites of extracellular substrates for each cathepsin are shown in parentheses.

## References

[1] Turk V., Stoka V., Vasiljeva O., Renko M., Sun T., Turk B., Turk D. (2012). Cysteine cathepsins: From structure, function and regulation to new frontiers. Biochim. Biophys. Acta.

[2] Turk V., Turk B., Turk D. (2001). Lysosomal cysteine proteases: Facts and opportunities. EMBO J..

[3] Rossi A., Deveraux Q., Turk B., Sali A. (2004). Comprehensive search for cysteine cathepsins in the human genome. Biol. Chem..

[4] Vidmar R., Vizovisek M., Turk D., Turk B., Fonovic M. (2017). Protease cleavage site *fingerprinting* by label-free in-gel degradomics reveals pH-dependent specificity switch of legumain. EMBO J..

[5] Vizovisek M., Vidmar R., Van Quickelberghe E., Impens F., Andjelkovic U., Sobotic B., Stoka V., Gevaert K., Turk B., Fonovic M. (2015). Fast profiling of protease specificity reveals similar substrate specificities for cathepsins K, L and S. Proteomics.

[6] Biniossek M.L., Nagler D.K., Becker-Pauly C., Schilling O. (2011). Proteomic identification of protease cleavage sites characterizes prime and non-prime specificity of cysteine cathepsins B, L, and S. J. Proteome Res..

[7] Choe Y., Leonetti F., Greenbaum D.C., Lecaille F., Bogyo M., Bromme D., Ellman J.A., Craik C.S. (2006). Substrate profiling of cysteine proteases using a combinatorial peptide library identifies functionally unique specificities. J. Biol. Chem..

[8] Brix K., Dunkhorst A., Mayer K., Jordans S. (2008). Cysteine cathepsins: Cellular roadmap to different functions. Biochimie.

[9] Brix K., Linke M., Tepel C., Herzog V. (2001). Cysteine proteinases mediate extracellular prohormone processing in the thyroid. Biol. Chem..

[10] Kramer L., Turk D., Turk B. (2017). The Future of Cysteine Cathepsins in Disease Management. Trends Pharm. Sci..

[11] Vasiljeva O., Reinheckel T., Peters C., Turk D., Turk V., Turk B. (2007). Emerging roles of cysteine cathepsins in disease and their potential as drug targets. Curr. Pharm. Des..

[12] Reiser J., Adair B., Reinheckel T. (2010). Specialized roles for cysteine cathepsins in health and disease. J. Clin. Investig..

[13] Gocheva V., Joyce J.A. (2007). Cysteine cathepsins and the cutting edge of cancer invasion. Cell Cycle.

[14] Bromme D., Wilson S., Parks W.C., Mecham R.P. (2011). Role of Cysteine Cathepsins in Extracellular Proteolysis. Extracellular Matrix Degradation.

[15] Vizovisek M., Fonovic M., Turk B. (2019). Cysteine cathepsins in extracellular matrix remodeling: Extracellular matrix degradation and beyond. Matrix Biol..

[16] Repnik U., Starr A.E., Overall C.M., Turk B. (2015). Cysteine Cathepsins Activate ELR Chemokines and Inactivate Non-ELR Chemokines. J. Biol. Chem..

[17] Ainscough J.S., Macleod T., McGonagle D., Brakefield R., Baron J.M., Alase A., Wittmann M., Stacey M. (2017). Cathepsin S is the major activator of the psoriasis-associated proinflammatory cytokine IL-36γ. Proc. Natl. Acad. Sci. USA.

[18] Breznik B., Motaln H., Lah Turnsek T. (2017). Proteases and cytokines as mediators of interactions between cancer and stromal cells in tumours. Biol. Chem..

[19] Ohashi K., Naruto M., Nakaki T., Sano E. (2003). Identification of interleukin-8 converting enzyme as cathepsin L.. Biochim. Biophys. Acta.

[20] Hira V.V., Verbovsek U., Breznik B., Srdic M., Novinec M., Kakar H., Wormer J., der Swaan B.V., Lenarcic B., Juliano L. (2017). Cathepsin K cleavage of SDF-1alpha inhibits its chemotactic activity towards glioblastoma stem-like cells. Biochim. Biophys. Acta Mol. Cell Res..

[21] Sobotic B., Vizovisek M., Vidmar R., Van Damme P., Gocheva V., Joyce J.A., Gevaert K., Turk V., Turk B., Fonovic M. (2015). Proteomic Identification of Cysteine Cathepsin Substrates Shed from the Surface of Cancer Cells. Mol. Cell. Proteom..

[22] Clark A.K., Grist J., Al-Kashi A., Perretti M., Malcangio M. (2012). Spinal cathepsin S and fractalkine contribute to chronic pain in the collagen-induced arthritis model. Arthritis Rheum..

[23] Clark A.K., Yip P.K., Malcangio M. (2009). The liberation of fractalkine in the dorsal horn requires microglial cathepsin S. J. Neurosci..

[24] Turk D., Guncar G., Podobnik M., Turk B. (1998). Revised definition of substrate binding sites of papain-like cysteine proteases. Biol. Chem..

[25] Turk V., Stoka V., Turk D. (2008). Cystatins: Biochemical and structural properties, and medical relevance. Front. Biosci..

[26] Turk B., Turk D., Salvesen G.S. (2002). Regulating cysteine protease activity: Essential role of protease inhibitors as guardians and regulators. Curr. Pharm. Des..

[27] Drake F.H., Dodds R.A., James I.E., Connor J.R., Debouck C., Richardson S., Lee-Rykaczewski E., Coleman L., Rieman D., Barthlow R. (1996). Cathepsin K, but not cathepsins B, L, or S, is abundantly expressed in human osteoclasts. J. Biol. Chem..

[28] Riese R.J., Mitchell R.N., Villadangos J.A., Shi G.P., Palmer J.T., Karp E.R., De Sanctis G.T., Ploegh H.L., Chapman H.A. (1998). Cathepsin S activity regulates antigen presentation and immunity. J. Clin. Investig..

[29] Nakagawa T.Y., Brissette W.H., Lira P.D., Griffiths R.J., Petrushova N., Stock J., McNeish J.D., Eastman S.E., Howard E.D., Clarke S.R. (1999). Impaired invariant chain degradation and antigen presentation and diminished collagen-induced arthritis in cathepsin S null mice. Immunity.

[30] Bromme D., Li Z., Barnes M., Mehler E. (1999). Human cathepsin V functional expression, tissue distribution, electrostatic surface potential, enzymatic characterization, and chromosomal localization. Biochemistry.

[31] Shi G.P., Villadangos J.A., Dranoff G., Small C., Gu L., Haley K.J., Riese R., Ploegh H.L., Chapman H.A. (1999). Cathepsin S required for normal MHC class II peptide loading and germinal center development. Immunity.

[32] Wex T., Buhling F., Wex H., Gunther D., Malfertheiner P., Weber E., Bromme D. (2001). Human cathepsin W, a cysteine protease predominantly expressed in NK cells, is mainly localized in the endoplasmic reticulum. J. Immunol..

[33] Guncar G., Pungercic G., Klemencic I., Turk V., Turk D. (1999). Crystal structure of MHC class II-associated p41 Ii fragment bound to cathepsin L reveals the structural basis for differentiation between cathepsins L and S. EMBO J..

[34] Turk B., Dolenc I., Turk V., Bieth J.G. (1993). Kinetics of the pH-induced inactivation of human cathepsin L.. Biochemistry.

[35] Turk B., Bieth J.G., Bjork I., Dolenc I., Turk D., Cimerman N., Kos J., Colic A., Stoka V., Turk V. (1995). Regulation of the activity of lysosomal cysteine proteinases by pH-induced inactivation and/or endogenous protein inhibitors, cystatins. Biol. Chem. Hoppe Seyler.

[36] Kirschke H., Wiederanders B., Bromme D., Rinne A. (1989). Cathepsin S from bovine spleen. Purification, distribution, intracellular localization and action on proteins. Biochem. J..

[37] Bromme D., Okamoto K., Wang B.B., Biroc S. (1996). Human cathepsin O2, a matrix protein-degrading cysteine protease expressed in osteoclasts. Functional expression of human cathepsin O2 in Spodoptera frugiperda and characterization of the enzyme. J. Biol. Chem..

[38] Almeida P.C., Nantes I.L., Rizzi C.C., Júdice W.A., Chagas J.R., Juliano L., Nader H.B., Tersariol I.L. (1999). Cysteine proteinase activity regulation. A possible role of heparin and heparin-like glycosaminoglycans. J. Biol. Chem..

[39] Caglic D., Pungercar J.R., Pejler G., Turk V., Turk B. (2007). Glycosaminoglycans facilitate procathepsin B activation through disruption of propeptide-mature enzyme interactions. J. Biol. Chem..

[40] Li Z., Yasuda Y., Li W., Bogyo M., Katz N., Gordon R.E., Fields G.B., Bromme D. (2004). Regulation of collagenase activities of human cathepsins by glycosaminoglycans. J. Biol. Chem..

[41] Yasuda Y., Li Z., Greenbaum D., Bogyo M., Weber E., Brömme D. (2004). Cathepsin V, a novel and potent elastolytic activity expressed in activated macrophages. J. Biol. Chem..

[42] Turk B. (2006). Targeting proteases: Successes, failures and future prospects. Nat. Rev. Drug Discov..

[43] Friedrichs B., Tepel C., Reinheckel T., Deussing J., von Figura K., Herzog V., Peters C., Saftig P., Brix K. (2003). Thyroid functions of mouse cathepsins B, K, and L. J. Clin. Investig..

[44] Sukhova G.K., Shi G.P., Simon D.I., Chapman H.A., Libby P. (1998). Expression of the elastolytic cathepsins S and K in human atheroma and regulation of their production in smooth muscle cells. J. Clin. Investig..

[45] Jordans S., Jenko-Kokalj S., Kühl N.M., Tedelind S., Sendt W., Brömme D., Turk D., Brix K. (2009). Monitoring compartment-specific substrate cleavage by cathepsins B, K, L, and S at physiological pH and redox conditions. BMC Biochem..

[46] Godat E., Hervé-Grvépinet V., Veillard F., Lecaille F., Belghazi M., Brömme D., Lalmanach G. (2008). Regulation of cathepsin K activity by hydrogen peroxide. Biol. Chem..

[47] Rozhin J., Sameni M., Ziegler G., Sloane B.F. (1994). Pericellular pH affects distribution and secretion of cathepsin B in malignant cells. Cancer Res..

[48] Olson O.C., Joyce J.A. (2015). Cysteine cathepsin proteases: Regulators of cancer progression and therapeutic response. Nat. Rev. Cancer.

[49] Wu H., Du Q., Dai Q., Ge J., Cheng X. (2017). Cysteine Protease Cathepsins in Atherosclerotic Cardiovascular Diseases. J. Atheroscler. Thromb..

[50] Nanda A., Gukovskaya A., Tseng J., Grinstein S. (1992). Activation of vacuolar-type proton pumps by protein kinase C. Role in neutrophil pH regulation. J. Biol. Chem..

[51] Reddy V.Y., Zhang Q.Y., Weiss S.J. (1995). Pericellular mobilization of the tissue-destructive cysteine proteinases, cathepsins B, L, and S, by human monocyte-derived macrophages. Proc. Natl. Acad. Sci. USA.

[52] Dames P., Zimmermann B., Schmidt R., Rein J., Voss M., Schewe B., Walz B., Baumann O. (2006). cAMP regulates plasma membrane vacuolar-type H+-ATPase assembly and activity in blowfly salivary glands. Proc. Natl. Acad. Sci. USA.

[53] Wang J., Cheng X., Xiang M.X., Alanne-Kinnunen M., Wang J.A., Chen H., He A., Sun X., Lin Y., Tang T.T. (2011). IgE stimulates human and mouse arterial cell apoptosis and cytokine expression and promotes atherogenesis in Apoe^−^/^−^ mice. J. Clin. Investig..

[54] Mohamed M.M., Sloane B.F. (2006). Cysteine cathepsins: Multifunctional enzymes in cancer. Nat. Rev. Cancer.

[55] Konttinen Y.T., Mandelin J., Li T.F., Salo J., Lassus J., Liljeström M., Hukkanen M., Takagi M., Virtanen I., Santavirta S. (2002). Acidic cysteine endoproteinase cathepsin K in the degeneration of the superficial articular hyaline cartilage in osteoarthritis. Arthritis Rheum..

[56] Naghavi M., John R., Naguib S., Siadaty M.S., Grasu R., Kurian K.C., van Winkle W.B., Soller B., Litovsky S., Madjid M. (2002). pH Heterogeneity of human and rabbit atherosclerotic plaques; a new insight into detection of vulnerable plaque. Atherosclerosis.

[57] Settembre C., Di Malta C., Polito V.A., Garcia Arencibia M., Vetrini F., Erdin S., Erdin S.U., Huynh T., Medina D., Colella P. (2011). TFEB links autophagy to lysosomal biogenesis. Science.

[58] Yan D., Wang H.W., Bowman R.L., Joyce J.A. (2016). STAT3 and STAT6 Signaling Pathways Synergize to Promote Cathepsin Secretion from Macrophages via IRE1α Activation. Cell Rep..

[59] Kreuzaler P.A., Staniszewska A.D., Li W., Omidvar N., Kedjouar B., Turkson J., Poli V., Flavell R.A., Clarkson R.W., Watson C.J. (2011). Stat3 controls lysosomal-mediated cell death in vivo. Nat. Cell Biol..

[60] Caglič D., Repnik U., Jedeszko C., Kosec G., Miniejew C., Kindermann M., Vasiljeva O., Turk V., Wendt K.U., Sloane B.F. (2013). The proinflammatory cytokines interleukin-1α and tumor necrosis factor α promote the expression and secretion of proteolytically active cathepsin S from human chondrocytes. Biol. Chem..

[61] Mohamed M.M., Cavallo-Medved D., Rudy D., Anbalagan A., Moin K., Sloane B.F. (2010). Interleukin-6 increases expression and secretion of cathepsin B by breast tumor-associated monocytes. Cell. Physiol. Biochem..

[62] Troen B.R. (2006). The regulation of cathepsin K gene expression. Ann. N. Y. Acad. Sci..

[63] Ruettger A., Schueler S., Mollenhauer J.A., Wiederanders B. (2007). Cathepsins B, K, and L are regulated by a defined collagen type II peptide via activation of classical protein kinase C and p38 MAP kinase in articular chondrocytes. J. Biol. Chem..

[64] Hashimoto Y., Kondo C., Katunuma N. (2015). An Active 32-kDa Cathepsin L Is Secreted Directly from HT 1080 Fibrosarcoma Cells and Not via Lysosomal Exocytosis. PLoS ONE.

[65] Rodríguez A., Webster P., Ortego J., Andrews N.W. (1997). Lysosomes behave as Ca^2+^-regulated exocytic vesicles in fibroblasts and epithelial cells. J. Cell Biol..

[66] Ichinose S., Usuda J., Hirata T., Inoue T., Ohtani K., Maehara S., Kubota M., Imai K., Tsunoda Y., Kuroiwa Y. (2006). Lysosomal cathepsin initiates apoptosis, which is regulated by photodamage to Bcl-2 at mitochondria in photodynamic therapy using a novel photosensitizer, ATX-s10 (Na). Int. J. Oncol..

[67] Chwieralski C.E., Welte T., Bühling F. (2006). Cathepsin-regulated apoptosis. Apoptosis.

[68] Cheng X.W., Kuzuya M., Nakamura K., Di Q., Liu Z., Sasaki T., Kanda S., Jin H., Shi G.P., Murohara T. (2006). Localization of cysteine protease, cathepsin S, to the surface of vascular smooth muscle cells by association with integrin alphanubeta3. Am. J. Pathol..

[69] Obermajer N., Svajger U., Bogyo M., Jeras M., Kos J. (2008). Maturation of dendritic cells depends on proteolytic cleavage by cathepsin X. J. Leukoc. Biol..

[70] Obermajer N., Premzl A., Zavasnik Bergant T., Turk B., Kos J. (2006). Carboxypeptidase cathepsin X mediates beta2-integrin-dependent adhesion of differentiated U-937 cells. Exp. Cell Res..

[71] Nascimento F.D., Rizzi C.C., Nantes I.L., Stefe I., Turk B., Carmona A.K., Nader H.B., Juliano L., Tersariol I.L. (2005). Cathepsin X binds to cell surface heparan sulfate proteoglycans. Arch. Biochem. Biophys..

[72] Sloane B.F., Rozhin J., Johnson K., Taylor H., Crissman J.D., Honn K.V. (1986). Cathepsin B: Association with plasma membrane in metastatic tumors. Proc. Natl. Acad. Sci. USA.

[73] Mai J., Finley R.L., Waisman D.M., Sloane B.F. (2000). Human procathepsin B interacts with the annexin II tetramer on the surface of tumor cells. J. Biol. Chem..

[74] Cavallo-Medved D., Dosescu J., Linebaugh B.E., Sameni M., Rudy D., Sloane B.F. (2003). Mutant K-ras regulates cathepsin B localization on the surface of human colorectal carcinoma cells. Neoplasia.

[75] Campo E., Muñoz J., Miquel R., Palacín A., Cardesa A., Sloane B.F., Emmert-Buck M.R. (1994). Cathepsin B expression in colorectal carcinomas correlates with tumor progression and shortened patient survival. Am. J. Pathol..

[76] Hazen L.G., Bleeker F.E., Lauritzen B., Bahns S., Song J., Jonker A., Van Driel B.E., Lyon H., Hansen U., Köhler A. (2000). Comparative localization of cathepsin B protein and activity in colorectal cancer. J. Histochem. Cytochem..

[77] Rozario T., DeSimone D.W. (2009). The extracellular matrix in development and morphogenesis: A dynamic view. Dev. Biol..

[78] Ozbek S., Balasubramanian P.G., Chiquet-Ehrismann R., Tucker R.P., Adams J.C. (2010). The evolution of extracellular matrix. Mol. Biol. Cell.

[79] Murphy-Ullrich J.E., Sage E.H. (2014). Revisiting the matricellular concept. Matrix Biol..

[80] Bonnans C., Chou J., Werb Z. (2014). Remodelling the extracellular matrix in development and disease. Nat. Rev. Mol. Cell Biol..

[81] Werb Z. (1997). ECM and cell surface proteolysis: Regulating cellular ecology. Cell.

[82] Fonovic M., Turk B. (2014). Cysteine cathepsins and extracellular matrix degradation. Biochim. Biophys. Acta.

[83] Apte S.S., Parks W.C. (2015). Metalloproteinases: A parade of functions in matrix biology and an outlook for the future. Matrix Biol..

[84] Liu C.L., Guo J., Zhang X., Sukhova G.K., Libby P., Shi G.P. (2018). Cysteine protease cathepsins in cardiovascular disease: From basic research to clinical trials. Nat. Rev. Cardiol..

[85] Nakao S., Zandi S., Sun D., Hafezi-Moghadam A. (2018). Cathepsin B-mediated CD18 shedding regulates leukocyte recruitment from angiogenic vessels. FASEB J..

[86] Mai J., Sameni M., Mikkelsen T., Sloane B.F. (2002). Degradation of extracellular matrix protein tenascin-C by cathepsin B: An interaction involved in the progression of gliomas. Biol. Chem..

[87] Prudova A., Gocheva V., Auf dem Keller U., Eckhard U., Olson O.C., Akkari L., Butler G.S., Fortelny N., Lange P.F., Mark J.C. (2016). TAILS N-Terminomics and Proteomics Show Protein Degradation Dominates over Proteolytic Processing by Cathepsins in Pancreatic Tumors. Cell Rep..

[88] Guinec N., Dalet-Fumeron V., Pagano M. (1993). “In vitro” study of basement membrane degradation by the cysteine proteinases, cathepsins B, B-like and L. Digestion of collagen IV, laminin, fibronectin, and release of gelatinase activities from basement membrane fibronectin. Biol. Chem. Hoppe Seyler.

[89] Gineyts E., Bonnet N., Bertholon C., Millet M., Pagnon-Minot A., Borel O., Geraci S., Bonnelye E., Croset M., Suhail A. (2018). The C-Terminal Intact Forms of Periostin (iPTN) Are Surrogate Markers for Osteolytic Lesions in Experimental Breast Cancer Bone Metastasis. Calcif. Tissue Int..

[90] Podgorski I., Linebaugh B.E., Koblinski J.E., Rudy D.L., Herroon M.K., Olive M.B., Sloane B.F. (2009). Bone marrow-derived cathepsin K cleaves SPARC in bone metastasis. Am. J. Pathol..

[91] Wang B., Sun J., Kitamoto S., Yang M., Grubb A., Chapman H.A., Kalluri R., Shi G.P. (2006). Cathepsin S controls angiogenesis and tumor growth via matrix-derived angiogenic factors. J. Biol. Chem..

[92] Du X., Chen N.L., Wong A., Craik C.S., Brömme D. (2013). Elastin degradation by cathepsin V requires two exosites. J. Biol. Chem..

[93] Fang W., He A., Xiang M.X., Lin Y., Wang Y., Li J., Yang C., Zhang X., Liu C.L., Sukhova G.K. (2018). Cathepsin K-deficiency impairs mouse cardiac function after myocardial infarction. J. Mol. Cell. Cardiol..

[94] Kehlet S.N., Bager C.L., Willumsen N., Dasgupta B., Brodmerkel C., Curran M., Brix S., Leeming D.J., Karsdal M.A. (2017). Cathepsin-S degraded decorin are elevated in fibrotic lung disorders—Development and biological validation of a new serum biomarker. BMC Pulm. Med..

[95] Clark A.K., Yip P.K., Grist J., Gentry C., Staniland A.A., Marchand F., Dehvari M., Wotherspoon G., Winter J., Ullah J. (2007). Inhibition of spinal microglial cathepsin S for the reversal of neuropathic pain. Proc. Natl. Acad. Sci. USA.

[96] Zhao P., Lieu T., Barlow N., Metcalf M., Veldhuis N.A., Jensen D.D., Kocan M., Sostegni S., Haerteis S., Baraznenok V. (2014). Cathepsin S causes inflammatory pain via biased agonism of PAR2 and TRPV4. J. Biol. Chem..

[97] Garsen M., Rops A.L., Dijkman H., Willemsen B., van Kuppevelt T.H., Russel F.G., Rabelink T.J., Berden J.H., Reinheckel T., van der Vlag J. (2016). Cathepsin L is crucial for the development of early experimental diabetic nephropathy. Kidney Int..

[98] Mort J.S., Magny M.C., Lee E.R. (1998). Cathepsin B: An alternative protease for the generation of an aggrecan ‘metalloproteinase’ cleavage neoepitope. Biochem. J..

[99] Mort J.S., Beaudry F., Théroux K., Emmott A.A., Richard H., Fisher W.D., Lee E.R., Poole A.R., Laverty S. (2016). Early cathepsin K degradation of type II collagen in vitro and in vivo in articular cartilage. Osteoarthr. Cartil..

[100] Hou W.S., Li Z., Büttner F.H., Bartnik E., Brömme D. (2003). Cleavage site specificity of cathepsin K toward cartilage proteoglycans and protease complex formation. Biol. Chem..

[101] Atley L.M., Mort J.S., Lalumiere M., Eyre D.R. (2000). Proteolysis of human bone collagen by cathepsin K: Characterization of the cleavage sites generating by cross-linked N-telopeptide neoepitope. Bone.

[102] Kafienah W., Brömme D., Buttle D.J., Croucher L.J., Hollander A.P. (1998). Human cathepsin K cleaves native type I and II collagens at the N-terminal end of the triple helix. Biochem. J..

[103] Baumgrass R., Williamson M.K., Price P.A. (1997). Identification of peptide fragments generated by digestion of bovine and human osteocalcin with the lysosomal proteinases cathepsin B, D, L, H, and S. J. Bone Min. Res..

[104] Bossard M.J., Tomaszek T.A., Thompson S.K., Amegadzie B.Y., Hanning C.R., Jones C., Kurdyla J.T., McNulty D.E., Drake F.H., Gowen M. (1996). Proteolytic activity of human osteoclast cathepsin K. Expression, purification, activation, and substrate identification. J. Biol. Chem..

[105] Kubler A., Larsson C., Luna B., Andrade B.B., Amaral E.P., Urbanowski M., Orandle M., Bock K., Ammerman N.C., Cheung L.S. (2016). Cathepsin K Contributes to Cavitation and Collagen Turnover in Pulmonary Tuberculosis. J. Infect. Dis..

[106] Sloane B.F., Dunn J.R., Honn K.V. (1981). Lysosomal cathepsin B: Correlation with metastatic potential. Science.

[107] Vasiljeva O., Papazoglou A., Kruger A., Brodoefel H., Korovin M., Deussing J., Augustin N., Nielsen B.S., Almholt K., Bogyo M. (2006). Tumor cell-derived and macrophage-derived cathepsin B promotes progression and lung metastasis of mammary cancer. Cancer Res..

[108] Joyce J.A., Baruch A., Chehade K., Meyer-Morse N., Giraudo E., Tsai F.Y., Greenbaum D.C., Hager J.H., Bogyo M., Hanahan D. (2004). Cathepsin cysteine proteases are effectors of invasive growth and angiogenesis during multistage tumorigenesis. Cancer Cell.

[109] Ruffell B., Affara N.I., Cottone L., Junankar S., Johansson M., DeNardo D.G., Korets L., Reinheckel T., Sloane B.F., Bogyo M. (2013). Cathepsin C is a tissue-specific regulator of squamous carcinogenesis. Genes Dev..

[110] Cox J.L. (2009). Cystatins and cancer. Front. Biosci..

[111] Akkari L., Gocheva V., Quick M.L., Kester J.C., Spencer A.K., Garfall A.L., Bowman R.L., Joyce J.A. (2016). Combined deletion of cathepsin protease family members reveals compensatory mechanisms in cancer. Genes Dev..

[112] Sevenich L., Schurigt U., Sachse K., Gajda M., Werner F., Muller S., Vasiljeva O., Schwinde A., Klemm N., Deussing J. (2010). Synergistic antitumor effects of combined cathepsin B and cathepsin Z deficiencies on breast cancer progression and metastasis in mice. Proc. Natl. Acad. Sci. USA.

[113] Akkari L., Gocheva V., Kester J.C., Hunter K.E., Quick M.L., Sevenich L., Wang H.W., Peters C., Tang L.H., Klimstra D.S. (2014). Distinct functions of macrophage-derived and cancer cell-derived cathepsin Z combine to promote tumor malignancy via interactions with the extracellular matrix. Genes Dev..

[114] Dennemarker J., Lohmuller T., Mayerle J., Tacke M., Lerch M.M., Coussens L.M., Peters C., Reinheckel T. (2010). Deficiency for the cysteine protease cathepsin L promotes tumor progression in mouse epidermis. Oncogene.

[115] Quail D.F., Joyce J.A. (2013). Microenvironmental regulation of tumor progression and metastasis. Nat. Med..

[116] Gocheva V., Zeng W., Ke D., Klimstra D., Reinheckel T., Peters C., Hanahan D., Joyce J.A. (2006). Distinct roles for cysteine cathepsin genes in multistage tumorigenesis. Genes Dev..

[117] Small D.M., Burden R.E., Jaworski J., Hegarty S.M., Spence S., Burrows J.F., McFarlane C., Kissenpfennig A., McCarthy H.O., Johnston J.A. (2013). Cathepsin S from both tumor and tumor-associated cells promote cancer growth and neovascularization. Int. J. Cancer.

[118] Vasiljeva O., Korovin M., Gajda M., Brodoefel H., Bojic L., Kruger A., Schurigt U., Sevenich L., Turk B., Peters C. (2008). Reduced tumour cell proliferation and delayed development of high-grade mammary carcinomas in cathepsin B-deficient mice. Oncogene.

[119] Bengsch F., Buck A., Gunther S.C., Seiz J.R., Tacke M., Pfeifer D., von Elverfeldt D., Sevenich L., Hillebrand L.E., Kern U. (2014). Cell type-dependent pathogenic functions of overexpressed human cathepsin B in murine breast cancer progression. Oncogene.

[120] Mitrovic A., Pecar Fonovic U., Kos J. (2017). Cysteine cathepsins B and X promote epithelial-mesenchymal transition of tumor cells. Eur. J. Cell Biol..

[121] Wang J., Chen L., Li Y., Guan X.Y. (2011). Overexpression of cathepsin Z contributes to tumor metastasis by inducing epithelial-mesenchymal transition in hepatocellular carcinoma. PLoS ONE.

[122] Tripathi R., Fiore L.S., Richards D.L., Yang Y., Liu J., Wang C., Plattner R. (2018). Abl and Arg mediate cysteine cathepsin secretion to facilitate melanoma invasion and metastasis. Sci. Signal..

[123] Gopinathan A., Denicola G.M., Frese K.K., Cook N., Karreth F.A., Mayerle J., Lerch M.M., Reinheckel T., Tuveson D.A. (2012). Cathepsin B promotes the progression of pancreatic ductal adenocarcinoma in mice. Gut.

[124] Maacha S., Hong J., von Lersner A., Zijlstra A., Belkhiri A. (2018). AXL Mediates Esophageal Adenocarcinoma Cell Invasion through Regulation of Extracellular Acidification and Lysosome Trafficking. Neoplasia.

[125] Rempel S.A., Rosenblum M.L., Mikkelsen T., Yan P.S., Ellis K.D., Golembieski W.A., Sameni M., Rozhin J., Ziegler G., Sloane B.F. (1994). Cathepsin B expression and localization in glioma progression and invasion. Cancer Res..

[126] Breznik B., Limback C., Porcnik A., Blejec A., Krajnc M.K., Bosnjak R., Kos J., Van Noorden C.J.F., Lah T.T. (2018). Localization patterns of cathepsins K and X and their predictive value in glioblastoma. Radiol. Oncol..

[127] Breznik B., Limbaeck Stokin C., Kos J., Khurshed M., Hira V.V.V., Bosnjak R., Lah T.T., Van Noorden C.J.F. (2018). Cysteine cathepsins B, X and K expression in peri-arteriolar glioblastoma stem cell niches. J. Mol. Histol..

[128] Shao G., Wang R., Sun A., Wei J., Peng K., Dai Q., Yang W., Lin Q. (2018). The E3 ubiquitin ligase NEDD4 mediates cell migration signaling of EGFR in lung cancer cells. Mol. Cancer.

[129] Vasiljeva O., Turk B. (2008). Dual contrasting roles of cysteine cathepsins in cancer progression: Apoptosis versus tumour invasion. Biochimie.

[130] Bruchard M., Mignot G., Derangere V., Chalmin F., Chevriaux A., Vegran F., Boireau W., Simon B., Ryffel B., Connat J.L. (2013). Chemotherapy-triggered cathepsin B release in myeloid-derived suppressor cells activates the Nlrp3 inflammasome and promotes tumor growth. Nat. Med..

[131] Butinar M., Prebanda M.T., Rajkovic J., Jeric B., Stoka V., Peters C., Reinheckel T., Kruger A., Turk V., Turk B. (2014). Stefin B deficiency reduces tumor growth via sensitization of tumor cells to oxidative stress in a breast cancer model. Oncogene.

[132] Zavrsnik J., Butinar M., Prebanda M.T., Krajnc A., Vidmar R., Fonovic M., Grubb A., Turk V., Turk B., Vasiljeva O. (2017). Cystatin C deficiency suppresses tumor growth in a breast cancer model through decreased proliferation of tumor cells. Oncotarget.

[133] Sevenich L., Bowman R.L., Mason S.D., Quail D.F., Rapaport F., Elie B.T., Brogi E., Brastianos P.K., Hahn W.C., Holsinger L.J. (2014). Analysis of tumour- and stroma-supplied proteolytic networks reveals a brain-metastasis-promoting role for cathepsin S. Nat. Cell Biol..

[134] Fukuda S., Schmid-Schonbein G.W. (2003). Regulation of CD18 expression on neutrophils in response to fluid shear stress. Proc. Natl. Acad. Sci. USA.

[135] Chang S.H., Kanasaki K., Gocheva V., Blum G., Harper J., Moses M.A., Shih S.C., Nagy J.A., Joyce J., Bogyo M. (2009). VEGF-A induces angiogenesis by perturbing the cathepsin-cysteine protease inhibitor balance in venules, causing basement membrane degradation and mother vessel formation. Cancer Res..

[136] Willumsen N., Bager C.L., Leeming D.J., Bay-Jensen A.C., Karsdal M.A. (2017). Nidogen-1 Degraded by Cathepsin S can be Quantified in Serum and is Associated with Non-Small Cell Lung Cancer. Neoplasia.

[137] Felbor U., Dreier L., Bryant R.A., Ploegh H.L., Olsen B.R., Mothes W. (2000). Secreted cathepsin L generates endostatin from collagen XVIII. EMBO J..

[138] Veillard F., Saidi A., Burden R.E., Scott C.J., Gillet L., Lecaille F., Lalmanach G. (2011). Cysteine cathepsins S and L modulate anti-angiogenic activities of human endostatin. J. Biol. Chem..

[139] Garnero P., Borel O., Byrjalsen I., Ferreras M., Drake F.H., McQueney M.S., Foged N.T., Delmas P.D., Delaissé J.M. (1998). The collagenolytic activity of cathepsin K is unique among mammalian proteinases. J. Biol. Chem..

[140] Yoshioka Y., Yamachika E., Nakanishi M., Ninomiya T., Nakatsuji K., Kobayashi Y., Fujii T., Iida S. (2018). Cathepsin K inhibitor causes changes in crystallinity and crystal structure of newly-formed mandibular bone in rats. Br. J. Oral Maxillofac. Surg..

[141] Sharma V., Panwar P., O’Donoghue A.J., Cui H., Guido R.V., Craik C.S., Brömme D. (2015). Structural requirements for the collagenase and elastase activity of cathepsin K and its selective inhibition by an exosite inhibitor. Biochem. J..

[142] Aguda A.H., Panwar P., Du X., Nguyen N.T., Brayer G.D., Brömme D. (2014). Structural basis of collagen fiber degradation by cathepsin K. Proc. Natl. Acad. Sci. USA.

[143] Panwar P., Butler G.S., Jamroz A., Azizi P., Overall C.M., Brömme D. (2017). Aging-associated modifications of collagen affect its degradation by matrix metalloproteinases. Matrix Biol..

[144] Yasuda Y., Kaleta J., Brömme D. (2005). The role of cathepsins in osteoporosis and arthritis: Rationale for the design of new therapeutics. Adv. Drug Deliv. Rev..

[145] Wheater G., Elshahaly M., Tuck S.P., Datta H.K., van Laar J.M. (2013). The clinical utility of bone marker measurements in osteoporosis. J. Transl. Med..

[146] Mort J.S., Recklies A.D., Poole A.R. (1984). Extracellular presence of the lysosomal proteinase cathepsin B in rheumatoid synovium and its activity at neutral pH. Arthritis Rheum..

[147] Hashimoto Y., Kakegawa H., Narita Y., Hachiya Y., Hayakawa T., Kos J., Turk V., Katunuma N. (2001). Significance of cathepsin B accumulation in synovial fluid of rheumatoid arthritis. Biochem. Biophys. Res. Commun..

[148] Pozgan U., Caglic D., Rozman B., Nagase H., Turk V., Turk B. (2010). Expression and activity profiling of selected cysteine cathepsins and matrix metalloproteinases in synovial fluids from patients with rheumatoid arthritis and osteoarthritis. Biol. Chem..

[149] Ben-Aderet L., Merquiol E., Fahham D., Kumar A., Reich E., Ben-Nun Y., Kandel L., Haze A., Liebergall M., Kosinska M.K. (2015). Detecting cathepsin activity in human osteoarthritis via activity-based probes. Arthritis Res. Ther..

[150] Weitoft T., Larsson A., Manivel V.A., Lysholm J., Knight A., Ronnelid J. (2015). Cathepsin S and cathepsin L in serum and synovial fluid in rheumatoid arthritis with and without autoantibodies. Rheumatology.

[151] Ruge T., Sodergren A., Wallberg-Jonsson S., Larsson A., Arnlov J. (2014). Circulating plasma levels of cathepsin S and L are not associated with disease severity in patients with rheumatoid arthritis. Scand. J. Rheumatol..

[152] Robert L., Robert A.M., Jacotot B. (1998). Elastin-elastase-atherosclerosis revisited. Atherosclerosis.

[153] Robert L., Robert A.M., Fülöp T. (2008). Rapid increase in human life expectancy: Will it soon be limited by the aging of elastin?. Biogerontology.

[154] Sukhova G.K., Zhang Y., Pan J.H., Wada Y., Yamamoto T., Naito M., Kodama T., Tsimikas S., Witztum J.L., Lu M.L. (2003). Deficiency of cathepsin S reduces atherosclerosis in LDL receptor-deficient mice. J. Clin. Investig..

[155] Samokhin A.O., Wong A., Saftig P., Bromme D. (2008). Role of cathepsin K in structural changes in brachiocephalic artery during progression of atherosclerosis in apoE-deficient mice. Atherosclerosis.

[156] Kitamoto S., Sukhova G.K., Sun J., Yang M., Libby P., Love V., Duramad P., Sun C., Zhang Y., Yang X. (2007). Cathepsin L deficiency reduces diet-induced atherosclerosis in low-density lipoprotein receptor-knockout mice. Circulation.

[157] Fonovic U.P., Jevnikar Z., Kos J. (2013). Cathepsin S generates soluble CX3CL1 (fractalkine) in vascular smooth muscle cells. Biol. Chem..

[158] Zhao C.F., Herrington D.M. (2016). The function of cathepsins B, D, and X in atherosclerosis. Am. J. Cardiovasc. Dis..

[159] Tohda C., Tohda M. (2017). Extracellular cathepsin L stimulates axonal growth in neurons. BMC Res. Notes.

[160] Tran A.P., Sundar S., Yu M., Lang B.T., Silver J. (2018). Modulation of Receptor Protein Tyrosine Phosphatase Sigma Increases Chondroitin Sulfate Proteoglycan Degradation through Cathepsin B Secretion to Enhance Axon Outgrowth. J. Neurosci..

[161] Saini M.G., Bix G.J. (2012). Oxygen-glucose deprivation (OGD) and interleukin-1 (IL-1) differentially modulate cathepsin B/L mediated generation of neuroprotective perlecan LG3 by neurons. Brain Res..

[162] Shen Y., Tenney A.P., Busch S.A., Horn K.P., Cuascut F.X., Liu K., He Z., Silver J., Flanagan J.G. (2009). PTPsigma is a receptor for chondroitin sulfate proteoglycan, an inhibitor of neural regeneration. Science.

[163] Moon H.Y., Becke A., Berron D., Becker B., Sah N., Benoni G., Janke E., Lubejko S.T., Greig N.H., Mattison J.A. (2016). Running-Induced Systemic Cathepsin B Secretion Is Associated with Memory Function. Cell Metab..

[164] Taggart C., Mall M.A., Lalmanach G., Cataldo D., Ludwig A., Janciauskiene S., Heath N., Meiners S., Overall C.M., Schultz C. (2017). Protean proteases: At the cutting edge of lung diseases. Eur. Respir. J..

[165] Perdereau C., Godat E., Maurel M.C., Hazouard E., Diot E., Lalmanach G. (2006). Cysteine cathepsins in human silicotic bronchoalveolar lavage fluids. Biochim. Biophys. Acta.

[166] Dongre A., Clements D., Fisher A.J., Johnson S.R. (2017). Cathepsin K in Lymphangioleiomyomatosis: LAM Cell-Fibroblast Interactions Enhance Protease Activity by Extracellular Acidification. Am. J. Pathol..

[167] Squeglia F., Ruggiero A., Berisio R. (2018). Collagen degradation in tuberculosis pathogenesis: The biochemical consequences of hosting an undesired guest. Biochem. J..

[168] Srivastava M., Steinwede K., Kiviranta R., Morko J., Hoymann H.G., Langer F., Buhling F., Welte T., Maus U.A. (2008). Overexpression of cathepsin K in mice decreases collagen deposition and lung resistance in response to bleomycin-induced pulmonary fibrosis. Respir. Res..

[169] Buhling F., Rocken C., Brasch F., Hartig R., Yasuda Y., Saftig P., Bromme D., Welte T. (2004). Pivotal role of cathepsin K in lung fibrosis. Am. J. Pathol..

[170] Kasabova M., Villeret B., Gombault A., Lecaille F., Reinheckel T., Marchand-Adam S., Couillin I., Lalmanach G. (2016). Discordance in cathepsin B and cystatin C expressions in bronchoalveolar fluids between murine bleomycin-induced fibrosis and human idiopathic fibrosis. Respir. Res..

[171] Small D.M., Brown R.R., Doherty D.F., Abladey A., Zhou-Suckow Z., Delaney R.J., Kerrigan L., Dougan C.M., Borensztajn K.S., Holsinger L. (2019). Targeting of Cathepsin S Reduces Cystic Fibrosis-like Lung Disease. Eur. Respir. J..

[172] Elmariah S.B., Reddy V.B., Lerner E.A. (2014). Cathepsin S signals via PAR2 and generates a novel tethered ligand receptor agonist. PLoS ONE.

[173] Taleb S., Cancello R., Clément K., Lacasa D. (2006). Cathepsin s promotes human preadipocyte differentiation: Possible involvement of fibronectin degradation. Endocrinology.

[174] Douglas S.A., Lamothe S.E., Singleton T.S., Averett R.D., Platt M.O. (2018). Human cathepsins K, L, and S: Related proteases, but unique fibrinolytic activity. Biochim. Biophys. Acta Gen. Subj..

[175] Ferrall-Fairbanks M.C., West D.M., Douglas S.A., Averett R.D., Platt M.O. (2018). Computational predictions of cysteine cathepsin-mediated fibrinogen proteolysis. Protein Sci..

[176] Ogasawara S., Cheng X.W., Inoue A., Hu L., Piao L., Yu C., Goto H., Xu W., Zhao G., Lei Y. (2017). Cathepsin K activity controls cardiotoxin-induced skeletal muscle repair in mice. J. Cachexia Sarcopenia Muscle.

[177] Lechner A.M., Assfalg-Machleidt I., Zahler S., Stoeckelhuber M., Machleidt W., Jochum M., Nagler D.K. (2006). RGD-dependent binding of procathepsin X to integrin alphavbeta3 mediates cell-adhesive properties. J. Biol. Chem..

[178] Sina C., Lipinski S., Gavrilova O., Aden K., Rehman A., Till A., Rittger A., Podschun R., Meyer-Hoffert U., Haesler R. (2013). Extracellular cathepsin K exerts antimicrobial activity and is protective against chronic intestinal inflammation in mice. Gut.

[179] Jennewein C., Tran N., Paulus P., Ellinghaus P., Eble J.A., Zacharowski K. (2011). Novel aspects of fibrin(ogen) fragments during inflammation. Mol. Med..

[180] Libert C. (2003). Inflammation: A nervous connection. Nature.

[181] Wolf Y., Yona S., Kim K.W., Jung S. (2013). Microglia, seen from the CX3CR1 angle. Front. Cell. Neurosci..

[182] Pislar A., Kos J. (2014). Cysteine cathepsins in neurological disorders. Mol. Neurobiol..

[183] Cocchiaro P., De Pasquale V., Della Morte R., Tafuri S., Avallone L., Pizard A., Moles A., Pavone L.M. (2017). The Multifaceted Role of the Lysosomal Protease Cathepsins in Kidney Disease. Front. Cell Dev. Biol..

[184] Sena B.F., Figueiredo J.L., Aikawa E. (2017). Cathepsin S As an Inhibitor of Cardiovascular Inflammation and Calcification in Chronic Kidney Disease. Front. Cardiovasc. Med..

[185] Kiviranta R., Morko J., Uusitalo H., Aro H.T., Vuorio E., Rantakokko J. (2001). Accelerated turnover of metaphyseal trabecular bone in mice overexpressing cathepsin K. J. Bone Miner. Res..

[186] Gowen M., Lazner F., Dodds R., Kapadia R., Feild J., Tavaria M., Bertoncello I., Drake F., Zavarselk S., Tellis I. (1999). Cathepsin K knockout mice develop osteopetrosis due to a deficit in matrix degradation but not demineralization. J. Bone Miner. Res..

[187] Mullard A. (2016). Merck &Co. drops osteoporosis drug odanacatib. Nat. Rev. Drug Discov..

[188] Bromme D., Panwar P., Turan S. (2016). Cathepsin K osteoporosis trials, pycnodysostosis and mouse deficiency models: Commonalities and differences. Expert Opin. Drug Discov..

[189] Panwar P., Soe K., Guido R.V., Bueno R.V., Delaisse J.M., Bromme D. (2016). A novel approach to inhibit bone resorption: Exosite inhibitors against cathepsin K. Br. J. Pharmacol..

[190] Panwar P., Xue L., Soe K., Srivastava K., Law S., Delaisse J.M., Bromme D. (2017). An Ectosteric Inhibitor of Cathepsin K Inhibits Bone Resorption in Ovariectomized Mice. J. Bone Miner. Res..

[191] Burden R.E., Gormley J.A., Jaquin T.J., Small D.M., Quinn D.J., Hegarty S.M., Ward C., Walker B., Johnston J.A., Olwill S.A. (2009). Antibody-mediated inhibition of cathepsin S blocks colorectal tumor invasion and angiogenesis. Clin. Cancer Res..

[192] Burden R.E., Gormley J.A., Kuehn D., Ward C., Kwok H.F., Gazdoiu M., McClurg A., Jaquin T.J., Johnston J.A., Scott C.J. (2012). Inhibition of Cathepsin S by Fsn0503 enhances the efficacy of chemotherapy in colorectal carcinomas. Biochimie.

[193] Sanman L.E., Bogyo M. (2014). Activity-based profiling of proteases. Annu. Rev. Biochem..

[194] Withana N.P., Saito T., Ma X., Garland M., Liu C., Kosuge H., Amsallem M., Verdoes M., Ofori L.O., Fischbein M. (2016). Dual-Modality Activity-Based Probes as Molecular Imaging Agents for Vascular Inflammation. J. Nucl. Med..

[195] Walker E., Mann M., Honda K., Vidimos A., Schluchter M.D., Straight B., Bogyo M., Popkin D., Basilion J.P. (2017). Rapid visualization of nonmelanoma skin cancer. J. Am. Acad. Derm..

[196] Withana N.P., Ma X., McGuire H.M., Verdoes M., van der Linden W.A., Ofori L.O., Zhang R., Li H., Sanman L.E., Wei K. (2016). Non-invasive Imaging of Idiopathic Pulmonary Fibrosis Using Cathepsin Protease Probes. Sci. Rep..

[197] Watzke A., Kosec G., Kindermann M., Jeske V., Nestler H.P., Turk V., Turk B., Wendt K.U. (2008). Selective activity-based probes for cysteine cathepsins. Angew. Chem. Int. Ed. Engl..

[198] Hu H.Y., Vats D., Vizovisek M., Kramer L., Germanier C., Wendt K.U., Rudin M., Turk B., Plettenburg O., Schultz C. (2014). In vivo imaging of mouse tumors by a lipidated cathepsin S substrate. Angew. Chem. Int. Ed. Engl..

[199] Kramer L., Renko M., Zavrsnik J., Turk D., Seeger M.A., Vasiljeva O., Grutter M.G., Turk V., Turk B. (2017). Non-invasive in vivo imaging of tumour-associated cathepsin B by a highly selective inhibitory DARPin. Theranostics.

[200] Garland M., Yim J.J., Bogyo M. (2016). A Bright Future for Precision Medicine: Advances in Fluorescent Chemical Probe Design and Their Clinical Application. Cell Chem. Biol..

[201] Fang Y., Du F., Xu Y., Meng H., Huang J., Zhang X., Lu W., Liu S., Yu J. (2015). Enhanced cellular uptake and intracellular drug controlled release of VESylated gemcitabine prodrug nanocapsules. Colloids Surf. B Biointerfaces.

[202] Zhang X., Tang K., Wang H., Liu Y., Bao B., Fang Y., Zhang X., Lu W. (2016). Design, Synthesis, and Biological Evaluation of New Cathepsin B-Sensitive Camptothecin Nanoparticles Equipped with a Novel Multifuctional Linker. Bioconj. Chem..

[203] Mikhaylov G., Klimpel D., Schaschke N., Mikac U., Vizovisek M., Fonovic M., Turk V., Turk B., Vasiljeva O. (2014). Selective targeting of tumor and stromal cells by a nanocarrier system displaying lipidated cathepsin B inhibitor. Angew. Chem. Int. Ed. Engl..

[204] Vizovisek M., Vidmar R., Drag M., Fonovic M., Salvesen G.S., Turk B. (2018). Protease Specificity: Towards In Vivo Imaging Applications and Biomarker Discovery. Trends Biochem. Sci..

